# Biomechanical traits of salt marsh vegetation are insensitive to future climate scenarios

**DOI:** 10.1038/s41598-022-25525-3

**Published:** 2022-12-08

**Authors:** Maike Paul, Christina Bischoff, Ketil Koop-Jakobsen

**Affiliations:** 1 Ludwig Franzius Institute of Hydraulic, Estuarine and Coastal Engineering, Leibniz University Hannover, Nienburger Str. 4, 30167 Hannover, Germany; 2 Helmholtz Centre for Polar and Marine Research, Wadden Sea Station, Alfred Wegener Institute, Hafenstraße 43, 25992 List/Sylt, Germany; 3 Marine Biological Laboratory, The Ecosystems Center, Woods Hole, MA USA

**Keywords:** Environmental impact, Ecosystem services

## Abstract

Salt marshes provide wave and flow attenuation, making them attractive for coastal protection. It is necessary to predict their coastal protection capacity in the future, when climate change will increase hydrodynamic forcing and environmental parameters such as water temperature and CO_2_ content. We exposed the European salt marsh species *Spartina anglica* and *Elymus athericus* to enhanced water temperature (+ 3°) and CO_2_ (800 ppm) levels in a mesocosm experiment for 13 weeks in a full factorial design. Afterwards, the effect on biomechanic vegetation traits was assessed. These traits affect the interaction of vegetation with hydrodynamic forcing, forming the basis for wave and flow attenuation. *Elymus athericus* did not respond to any of the treatments suggesting that it is insensitive to such future climate changes. *Spartina anglica* showed an increase in diameter and flexural rigidity, while Young’s bending modulus and breaking force did not differ between treatments. Despite some differences between the future climate scenario and present conditions, all values lie within the natural trait ranges for the two species. Consequently, this mesocosm study suggests that the capacity of salt marshes to provide coastal protection is likely to remain constantly high and will only be affected by future changes in hydrodynamic forcing.

## Introduction

Salt marshes are highly valuable coastal ecosystems along low lying intertidal coastlines around the world. They provide a variety of ecosystem services, including wave and flow reduction, which is relevant for coastal protection. Salt marshes reduce the hydrodynamic load on the coastal landscape and its coastal protective infrastructures, such as dikes, thus contributing to their durability and reducing their maintenance costs^1^. Moreover, marshes can have the ability to keep up with sea level rise, if sufficient sediment is supplied^2,3^, which enables them to provide their ecosystem services also in the future. As a result, their integration into coastal protection and management strategies is increasingly called for^4–6^. To do so, it is necessary to project the coastal protection capacity of salt marshes into the future, when climate change will affect a wide range of environmental parameters. In the coastal context, the most prominent climate change effects are sea-level rise and enhanced hydrodynamic forcing due to increased storminess^7^. However, increased air and water temperature and CO_2_ content may also impact plant growth^8,9^. Especially, increased CO_2_ content can lead to different responses in different plant species as it can lead to increased photosynthesis in C_3_ species, while the higher capacity of CO_2_ fixation in C_4_ species leads to less sensitivity to CO_2_ content^9,10^. Whether such differences in plant growth will lead to an outcompetition of C_3_ species over C_4_ species in salt marshes under future enhanced CO_2_ conditions, as suggested by Arp et al.^10^, remains to be seen. However, species development will also depend on their growth niches with respect to other environmental parameters such as salinity, flooding frequency and hydrodynamic forcing^11^.

Plants respond to non-optimal environmental conditions by either following a tolerance or avoidance strategy^12^. With respect to hydrodynamic forcing, these strategies are based on a plant’s stiffness, which can be expressed by flexural rigidity. This biomechanical trait integrates the effect of tissue composition and its geometrical arrangement around the stem’s central axis^13^. Plants following a tolerance strategy will have stiff stems that are very resistant to breakage while plants with an avoidance strategy will be highly flexible. This flexibility allows them to reconfigure under hydrodynamic forcing and thus avoid associated drag forces^14^. Both strategies can be observed in salt marsh vegetation depending on species^15^ and life stage^14^, and the transition between them can be fluid. Within the range of species-specific stiffness, plants are capable of adapting their flexural rigidity to site specific hydrodynamic forcing. This aspect of vegetation plasticity leads to lower flexural rigidity in locations with higher hydrodynamic forcing by either adjusting tissue composition^11^ or plant geometry^16,17^, thus reducing the drag forces acting on the plant.

In addition to driving a plant’s response to hydrodynamic forcing and thus being an integral part of its survival strategy^18^, flexural rigidity also determines a plant’s capacity to attenuate waves and flow and thus provide coastal protection. A plant with higher flexural rigidity will remain more upright and move less under hydrodynamic forcing and will thus pose more drag on the approaching flow than more flexible plants^19,20^. As a result, the moving water will lose momentum^21,22^. If the plant density is sufficiently high, the accumulated momentum loss will reduce overall flow velocity^23^ and/or wave energy^24^.

To predict these ecosystem services and thus incorporate them in coastal protection and management strategies, it is paramount to understand how future climate conditions may affect both elements of the underlying interaction, i.e. biomechanic vegetation traits and hydrodynamic forcing, and thus a salt marsh’s capacity to attenuate waves and flow. In this study, we focussed on the biomechanic vegetation traits, which are dependent on plant growth. Thus, any changes in plant growth due to changes in temperature and CO_2_ content may have a direct impact on the plant’s interaction with hydrodynamic forcing and the associated ecosystem services. To contribute to a better understanding of the changes in biomechanic vegetation traits under future climate conditions, we exposed the salt marsh species *Spartina anglica* (C_4_ species, *Spartina* hereafter) and *Elymus athericus* (C_3_ species, *Elymus* hereafter) to increased water temperature (+ 3 °C) and excess CO_2_ (800 ppm) content in a mesocosm experiment and assessed the effects of these environmental conditions on plant biomechanics, measuring stem diameter *d*, Young’s bending modulus *E*, flexural rigidity *J* and breaking force *F*_*max*_ at two heights above the base for *Spartina* (5 and 15 cm) and one height for *Elymus* (10 cm). Stem geometry did not allow for two measurements along the *Elymus* stem.

## Results

### Species comparison

**Figure 1:**
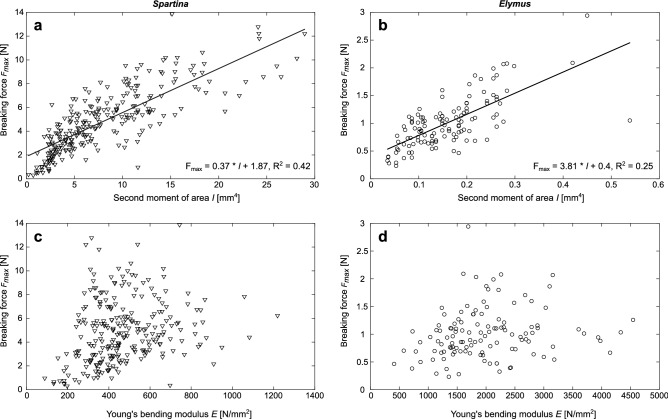
Relationship between breaking force *F*_*max*_ and vegetation traits. (**a**, **b**) relationship for the second moment of area *I*; (**c**, **d**) relationship for Young’s bending modulus *E.* Plots are provided for *Spartina* (**a**, **c**) and *Elymus* (**b**, **d**). Weak linear relationships are given for *I* but not for *E*. Note the different x-axes ranges for the two species.

Correlating traits with each other confirms that Young’s bending modulus as a proxy for material composition is independent of stem shape, i.e. outer diameter and second moment of area. The breaking force a stem can withstand weakly depends on stem shape but not on material composition (Fig. 1).

Comparing the traits of the two species shows clear differences in both plant morphology and material composition, irrespective of treatment. *Elymus* stems are statistically significantly thinner than *Spartina* stems at both measured heights (t-test, p < 0.01 for all treatments), while they have a higher Young’s bending modulus (t-test, p < 0.01 for all treatments, Table 1).

**Table 1:** Vegetation traits per sample type averaged across all treatments ± standard deviation.

		*Spartina* 5 cm (S5, n = 135)	*Spartina* 15 cm (S15, n = 120)	*Elymus* 10 cm (E10, n = 121)
Stem diameter *d*	mm	3.72 ± 0.54	3.09 ± 0.55	1.31 ± 0.17
Second moment of area *I*	mm^4^	10.54 ± 5.97	5.33 ± 3.83	0.16 ± 0.08
Young’s bending modulus *E*	N/mm^2^	470 ± 149	464 ± 209	1984 ± 793

Sample types are given with species and distance above the base with the abbreviation and sample size added in parentheses.

*Spartina* stems taper towards the tips, which is associated with changes in biomechanical traits along the stem^15^. Comparing the measurements 5 cm (S5) and 15 cm (S15) above the base within each treatment confirms this behaviour (Table 2). The stem diameter *d* and second moment of area *I* are significantly larger at S5 than at S15 at the 5% level across all treatments (t-test). The Young’s bending modulus *E* does not vary with height under the control and + 3 °C treatment, suggesting that material composition does not vary strongly along this part of the stem in these cases. Under the influence of added CO_2_, however, a statistically significant difference between the heights is observed, albeit with an unconclusive trend as values decrease with increasing height for the + CO_2_ treatment and increase for the + 3 °C/+ CO_2_ treatment (Table 2).

**Table 2:** Comparison of vegetation traits at 5 cm (S5) and 15 cm (S15) above the base along the *Spartina* stem per treatment.

Treatment	n	Mean ± standard deviation						p-value		
	S5/S15	*d* (S5)	*d* (S15)	*I* (S5)	*I* (S15)	*E* (S5)	*E* (S15)	*d*	*I*	*E*
Control	30/35	3.42 ± 0.49	2.92 ± 0.41	7.48 ± 4.00	4.00 ± 2.45	521.14 ± 141.29	459.87 ± 174.48	**< 0.001**	**< 0.001**	0.129
+ 3 °C	35/25	3.72 ± 0.47	2.94 ± 0.51	11.22 ± 4.89	4.29 ± 2.87	461.85 ± 131.18	471.46 ± 181.93	**< 0.001**	**< 0.001**	0.813
+ CO_2_	35/25	3.70 ± 0.48	3.09 ± 0.68	10.09 ± 5.29	5.66 ± 4.36	502.11 ± 175.79	362.67 ± 129.02	**< 0.001**	**< 0.001**	**0.001**
+ 3 °C/ + CO_2_	35/35	4.00 ± 0.57	3.37 ± 0.52	13.93 ± 7.40	7.19 ± 4.46	403.28 ± 118.11	534.96 ± 272.69	**< 0.001**	**< 0.001**	**0.011**

Mean values and standard deviation are given for *d* = diameter (mm), *I* = Second moment of area (mm^4^) and *E* = Young’s bending modulus (N/mm^2^) across all replicates per treatment with the given sample sizes n and p-values were derived with a 2 sample t-test. Differences that are statistically significant at the 5% level are displayed in bold.

### Treatment effect

**Figure 2:**
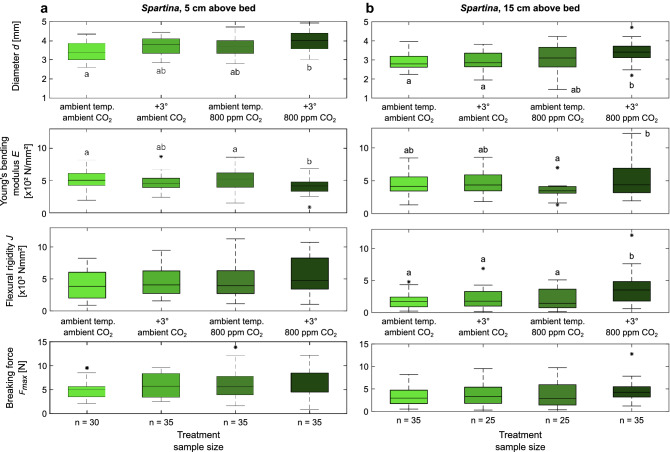
Comparison of vegetation traits between treatments and location along the stem for *Spartina*. (**a**) Measurements 5 cm above the base, (**b**) Measurements 15 cm above the base. Central marks of the boxplots indicate the median, boxes give inter-quartile range (IQR) and whiskers are maximum and minimum values within the 1.5 × IQR of the hinge. Outliers are indicated by an asterix each (*). Letters indicate results of Tukey–Kramer post-hoc Test, if statistically significant differences were identified. Sample sizes apply to all data in the respective columns.

When assessing the impact of the different treatments, no significant differences were found for any of the traits for *Elymus*. For *Spartina*, the influence of temperature (+ 3 °C) and CO_2_ (+ CO_2_) showed an increasing trend for stem diameter *d* as an indicator for morphology compared to the control. But only their combination (+ 3 °C/+ CO_2_) led to a statistically significant increase of stem diameter *d* (Fig. 2), which was irrespective of height above ground (S5: F = 7.14, p < 0.001; S15: F = 5.24, p = 0.002).

Young’s bending modulus *E* showed inconclusive results between S5 and S15. At S5 it decreased significantly for the + 3 °C/+ CO_2_ treatment compared to the control and + CO_2_ treatment, while the + 3 °C treatment differed from none of them (F = 4.43, p = 0.005, Fig. 2). And for S15, *E* increased for the + 3 °C/+ CO_2_ treatment but only differed significantly from the + CO_2_ treatment, for which *E* decreased compared to the other treatments (F = 3.53, p = 0.017, Fig. 2).

Flexural rigidity *J,* incorporating both the morphology as well as the plant material composition, showed an increase under the + 3 °C/+ CO_2_ treatment for S5 and S15 compared to the other treatments, but only for S15 was this increase statistically significant (S5: F = 1.89, p = 0.13; S15: F = 6.61, p < 0.001, Fig. 2). The breaking force *F*_*max*_ experienced during the bending test, and thus the force at which structural failure is experienced did not differ between treatments at all (Fig. 2).

## Discussion

The results show that neither *Spartina anglica* nor *Elymus athericus* are particularly sensitive to exposure to enhanced water temperature and CO_2_ content with respect to biomechanical plant traits, which show either no effects or an increase of the traits. After exposure to these conditions during the main growing phase, *Elymus* did not show any differences in stem diameter *d*, Young’s bending modulus *E*, flexural rigidity *J* and breaking force *F*_*max*_. For *Spartina*, some differences between the + 3 °C/+ CO_2_ treatment and the control could be found (Fig. 2), but the effect of the individual treatments remained inconclusive. Flexural rigidity *J*, as the main trait relevant for the plant’s interaction with hydrodynamics, was observed to increase statistically significant for the + 3 °C/+ CO_2_ treatment higher up along the stem (S15), while no significant change could be observed for S5. Consequently, along stem variation reduced during the + 3 °C/+ CO_2_ treatment. Such a response to future climate change would result in a more homogeneous bending behaviour making plant posture and thus interaction with waves and flow easier to predict^25^. The two plant species differ in their general stem geometry with *Elymus* showing a filled circular cross-section and *Spartina* showing a hollow circular cross-section. This distinct difference is reflected in the second moment of area *I* [Eqs. (2) and (3)] and thus accounted for in the calculation of flexural rigidity *J*. It may, however, affect breaking force *F*_*max*_ as it is known that hollow stems are more resistant to breaking than filled stems of the same outer diameter^13^. Nevertheless, it is not possible to isolate the effect of stem geometry on biomechanical plant traits in the present dataset, since the species differ in outer diameter and material composition (i.e. Young’s bending modulus *E*) which also have an influence on *F*_*max*_.

**Figure 3:**
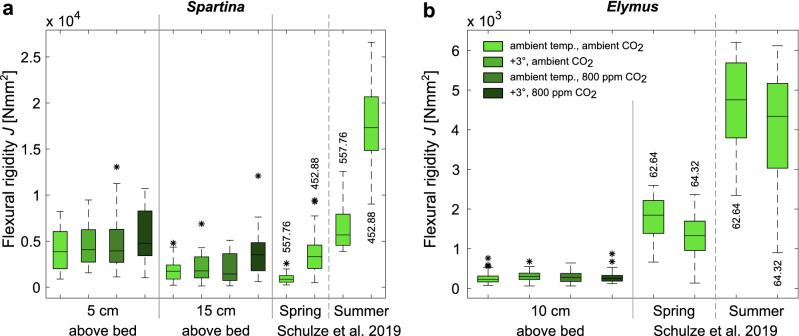
Comparison of flexural rigidity *J* from this study with data obtained from an undisturbed salt marsh^26^. Plots are provided for *Spartina* (**a**) and *Elymus* (**b**). Central marks of the boxplots indicate the median, boxes give inter-quartile range (IQR) and whiskers are maximum and minimum values within the 1.5 × IQR of the hinge. Outliers are indicated by an asterix each (*). Values above/below the boxplots indicate inundation time in h/year as reported by Schulze et al.^26^.

Overall, all values for flexural rigidity *J* still lie within the natural trait ranges found near the sampling site (Fig. 3a). For *Elymus* all values, including the control, remain at the lower limit of the values obtained by Schulze et al.^26^, but markedly lower than the values obtained in the same season, i.e. summer (Fig. 3b).

Given the close proximity of the two study locations (approx. 2.5 km apart) and thus comparable climatic and growth conditions prior to the experiment, this discrepancy in the control was unexpected. A possible explanation may be the exposure to saline water applied in this study. *Elymus athericus* grows in the mid to upper salt marsh where overall less saline conditions and less frequent flooding occur than in the pioneer zone. During the experiment, however, the mesocosm design did not allow for differentiated flooding, and both plant species were exposed to the same saline conditions and inundation frequency corresponding to conditions in the pioneer zone, since water was pumped directly from the sea to the mesocosms and inundation frequency could only be regulated for the entire mesocosm, which contained both *Spartina* and *Elymus* samples. Salinity stress has been found to negatively affect biomass production in *Elymus athericus*^8^. How it affects biomechanical plant traits, however, it not yet known. Flexural rigidity has been found to be negatively correlated with inundation frequency for *Spartina* and *Elymus* (Fig. 3^26^). More frequent inundations as consequence of climate change induced sea level rise are thus also likely to have an effect on biomechanical traits of *Elymus athericus* in the future, provided that *Elymus* adapts to such higher inundation frequencies and does not migrate to higher elevations.

With respect to the ecosystem services relevant for coastal protection, these results are promising. Flexural rigidity determines a plant’s resistance against bending under hydrodynamic forcing, which has implications for wave and flow attenuation^19,27,28^. If this trait remains unaffected by environmental conditions under a future climate, salt marsh vegetation will keep its high capacity to attenuate waves and flow^29^ and thus contribute to coastal protection. Equally encouraging is the observation that the breaking force *F*_*max*_ of neither of the species changed during the experiment. This suggests that plants may not become more susceptible to breaking in a future climate and therefore the number of stems providing wave and flow attenuation may not reduce due to their structural failure. However, the coastal protection capacity of salt marshes also depends on shoot density^30^ and the present experimental setup did not allow for an assessment of how this may change under the applied treatments.

The experiment was conducted for 13 weeks, covering the main growing season from early May to late August. This test duration was expected to be sufficiently long to detect potential changes as previous studies observed effects of increased atmospheric CO_2_ on plant growth and biomass production after approx. 10 weeks^8^. Enhanced atmospheric CO_2_ increases photosynthesis in C_3_ plants like *Elymus athericus,* thus leading to enhanced growth^8^, while this effect was observed to a lesser extent for C_4_ plants like *Spartina anglica*^31^. An enhanced biomass production, however, may result in lower biomechanical trait values, namely Young’s bending modulus *E* and flexural rigidity *J*, as it is likely to reduce lignin levels, which are paramount for the mechanical support of vascular plant tissue^32^. In this study, the water is likely to have assimilated large quantities of the added CO_2_, attenuating the enrichment of the mesocosm’s atmosphere, and thereby the enrichment’s contribution to photosynthesis.

An enhanced biomass production can also be expected as response to higher temperature levels for both C_3_ and C_4_ species^31^, however, this did not reflect in the biomechanical traits in this study. A lack of difference between the treatments with respect to temperature may be due to the mesocosm design. The temperature increase was simulated by heating the water before entering the mesocosms while their roofs were kept closed most of the time to preserve the CO_2_ enrichment, which resulted in a heating of the aboveground atmosphere in the mesocosms. The applied temperature control specifically targeted the water temperature and the temperature effect therefore specifically reflects a temperature difference experienced constantly by the root system. It is, however, likely that the overall heating of the mesocosms’ atmosphere had a stronger impact on plant growth than the + 3 °C water temperature increase in the respective treatments. In general, this reflects a fundamental challenge when setting up large scale mesocosms outdoors based on natural lighting. A closed headspace of the mesocosms is needed to maintain the CO_2_ enrichment, and under natural light conditions, heating is inevitable. Better temperature control can be maintained indoors, but it is often a trade-off with natural lighting and mesocosms size, which is significantly smaller indoors.

Nevertheless, the results suggest that salt marshes have the potential to keep their coastal protection capacity in the future. However, the efficiency at which the associated ecosystem services will be provided also depends on the local hydrodynamic regime, including water level and wave exposition, which is likely to change. While salt marshes may be able to adapt to sea level rise by vertical growth^2,33,34^, climate change induced sea level rise will also affect tidal water levels and storm surges^35,36^. Modelling of local effects of a 2 m global sea level rise suggest for instance an increased tidal range of up to 0.6 m in the German Bight^37^ which will result in higher flow velocities as the increased volume of water will have to flood and retreat within the constant timeframe of the tidal cycle. Enhanced water levels due to storm surge are driven by a combination of morphological features and storm intensity^38^ and hence strongly depend on the angle between coastline and storm direction^39^ with highest surges when storms approach perpendicular to the shore. In the German Bight, storms may occur more often from south-westerly to westerly directions in the future^40^ leading to an increase in intensity as well as frequency of extreme sea levels^38^ along the North Frisian coast line. As a result of these elevated water levels higher waves will reach the salt marsh as maximum wave height depends on water depth^41^. Salt marshes have been observed to significantly attenuate waves under storm surge conditions in a laboratory setting^29^, but how they will persist and how effective their coastal protection ecosystem services will be under such enhanced hydrodynamic forcing is still open to investigation. Particularly relevant will be in this context, how the interaction between biomechanical traits and hydrodynamic forcing will develop, given that an assessment of longer-term effects on marsh biomechanical traits, i.e. over multiple years and under exposure to a full combination of climate change impacts, is still pending.

## Methods

### Experimental design

To investigate how plant biomechanic traits will be affected by future levels of water temperature and CO_2_ content, salt marsh vegetation was exposed for 13 weeks to elevated levels of these environmental parameters in 12 large-scale outdoor mesocosms (diameter 1.8 m) in a full factorial design with true replication (n = 3). The mesocosm infrastructure is described and illustrated in Pansch et al.^42^. Live plants of the C_4_ species *Spartina anglica* and the C_3_ species *Elymus athericus*, representing typical vegetation of the pioneer zone (i.e. the marsh area below mean high water) and mid to high marsh, respectively, were collected at Hamburger Hallig (54°36′08, 08°49′09) in the German Wadden Sea in May 2021. Permission for collection of the plants was obtained from the German Wadden Sea Nationalpark authorities in compliance with national and international legislation. The plant species used in these experiments are not endangered.

Blocks of vegetated marsh (50 × 35 × 25 cm) were excavated and placed in skeleton folding boxes equipped with a water-permeable mesh-lining. They were directly transported to the Wadden Sea station of Alfred-Wegner-Institute—Helmholtz Centre for Polar and Marine Research (AWI) in List/Sylt, and placed in flow-through mesocosms with control over water temperature and CO_2_ content and set up to mimic tidal inundation. Three replicates of three different climate change scenarios and a control under ambient conditions were setup, resulting in 12 mesocosms containing plants of both species in separate containers. Scenarios included an increase in water temperature by 3 °C, an increase in CO_2_ up to 800 ppm and a combination of the two (Table 3). The elevated CO_2_ (800 ppm) and temperature (+ 3 °C) levels used in this experiment reflect the levels predicted to be reached by 2100 according to the Bern carbon cycle-climate model; one of the models used by the IPCC^43^. Salt water was pumped directly from the North Sea and heated up for treatment + 3 °C and + 3 °C/+ CO_2_, while for treatments + CO_2_ and + 3 °C/+ CO_2_, air enriched with CO_2_ to a constant concentration of 800 ppm was continuously pumped in the mesocosms which were covered by a transparent lid to prevent air escape. This led to constant differences compared to the control albeit variations over time were possible. At the beginning of the experiment, the CO_2_ enriched air was tested at the entry point confirming the 800 ppm level. During the experiment CO_2_ was sporadically monitored on 3 independent days in the air phase of CO_2_ enriched mesocosms. The concentrations were 50–150 ppm above the ambient concentrations, which showed values around 380 ppm. High humidity in the air-phase of the mesocosms rapidly damaged the IR-based CO_2_ sensors, which prevented continuous monitoring of CO_2_ in the air. CO_2_ removal in the air is attributed to CO_2_ absorption by water in mesocosm and soils, due to the carbonic acid equilibrium converting CO_2_ to HCO_3_^−^ (e.g.^42^), and to CO_2_ assimilation during photosynthetic uptake by the vegetation.

**Table 3:** Climate change treatments established in mesocosms.

Treatment	Water temperature	CO_2_ content
Control	Ambient	Ambient
+ 3 °C	+ 3 °C	Ambient
+ CO_2_	Ambient	800 ppm
+ 3 °C/+ CO_2_	+ 3 °C	800 ppm

Each treatment was replicated three times (n = 3) in independent mesocosms.

To simulate the tidal regime, the vegetation was placed on a moveable platform and lowered to be submerged for two hours once a day. To maintain the CO_2_ content in the air enclosed in the mesocosms, their lids remained closed and were only briefly opened for daily cleaning routines. As a result, air temperature fluctuated due to solar radiation entering through the transparent lid. Attempts to measure air temperature inside the mesocosms failed due to poor sensor performance. However, it is assumed that the effect is the same for all mesocosms given their placement next to each other.

### Sample preparation for measurements of biomechanic traits

At the end of the 13 week treatment in late August 2021, intact vegetation sods (17 × 24 cm) were removed from the mesocosms and transported to a cold room (+ 4 °C) in Hannover within 24 h. The following measurements were then performed within 5 days. For each species, treatment and replicate combination at least 10 specimen were chosen randomly and cut at soil level. Leaves were carefully removed with a knife and stem sections were prepared for measurement. For *Spartina*, two sections of 10 cm each were cut starting at the base to enable assessment of along-stem variations. A uniform distribution of biomechanical traits will cause homogeneous continuous bending, while along-stem variations can lead to vertical sectioning of bending angles inducing horizontal layers of different flow regimes^25^, which will have additional effects on wave and flow attenuation. For *Elymus* one section of 10 cm was cut starting 5 cm from the base. Cutting the same sections for *Elymus* than for *Spartina* was not feasible due to the geometry of the *Elymus* stems. As measurements of biomechanical traits were performed in the middle of these sections, this resulted in data for three sample types per treatment: *Spartina* at 5 and 15 cm (S5 and S15) and *Elymus* at 10 cm (E10) above the base, respectively.

### Calculation of biomechanical trait values

Biomechanical traits were estimated using a three-point bending test performed with a universal testing machine (ZwickRoell) using a 5 N load cell. A stamp was lowered onto the centre of the sample, resting on two support bars, with a displacement rate of 10 mm/min until the sample broke or buckled irreversibly to record the breaking force F_max_ (N), which the sample can withstand. For *Spartina*, the span width *s* (mm) between support bars was adjusted to keep a diameter to distance ratio between 1:10 and 1:15 while minimising the number of span width changes during measurements. For *Elymus* a constant span width of 28 mm was set, which exceeded these limits, but was the minimum realisable span width. The linear part of the recorded force–deflection curve was then used to calculate flexural rigidity *J* (N mm^2^):

1 $$J=\frac{{s}^{3}F}{48D}$$

with F = applied force (N) and D = resulting displacement (mm).

Flexural rigidity *J* is the product of Young’s bending modulus *E* (N/mm^2^) and second moment of area *I* (mm^4^). Young’s bending modulus *E* is a material property independent of a sample’s size and shape and thus considers the effect of plant material composition on its stiffness. A relation to biological tissue composition, i.e. CNP content, is possible^16^, but was not part of this study. The second moment of area *I* describes the stem’s geometrical arrangement around its central axis and can be derived from stem diameter *d*, assuming an idealised cross-sectional shape. For *Spartina* a hollow tubular cross-section [Eq. (2)] is assumed while for *Elymus* a filled circular cross-sections [Eq. (3)] is applied.

2 $$I=\frac{\pi \left({d}_{o}^{4}-{d}_{i}^{4}\right)}{64}$$

3 $$I=\frac{\pi {d}_{o}^{4}}{64}$$

with *d*_*o*_ = stem or outer diameter (mm) and *d*_*i*_ = inner diameter (mm). Together with values for flexural rigidity *J*, this allowed estimation of Young’s bending modulus *E* as an indicator for internal plant structure. Sample stem or outer diameter *d*_*o*_ was measured with a digital calliper gauge at four locations per sample and consecutively averaged following the approach of Miler et al.^44^. Inner diameter *d*_*i*_ was measured at the sample ends. In cases where the inner diameter could not be measured with the calliper gauge, *d*_*i*_ = 0.1 mm was assumed.

### Data analysis

Due to technical issues, data for S15 for one replicate of treatment + 3 °C and + CO_2_, respectively, could not be recorded. Consequently, a total of 255 *Spartina* and 121 *Elymus* samples were analysed. The software testXpertIII (V1.51, ZwickRoell) was used to compute Young’s bending modulus based on the recorded force–deflection curves and measured diameters. All other calculations and statistical analyses were then performed in Matlab (version R2019a, http://mathworks.com). All datasets were normally distributed following a One-sample Kolmogorov–Smirnov test and hence parametric tests were applied. Analysis of trait relationships was performed across the whole dataset, pooling all treatments as no differences were observed. Replicate comparison within treatments was analysed using a one way ANOVA. The data showed that differences occurred between two replicates of the same treatment with the third replicate usually spanning the whole range and no systematic pattern could be found between replicates of the same treatments with respect to any of the traits. It is thus hypothesised that the observed differences are likely due to large natural variability of the traits in combination with a relatively small sample size (n < 15) in all cases. Consequently, the data is pooled across replicates for further analysis. Species and height comparisons are performed using 2 sample t-tests and treatment comparison is analysed with a one way ANOVA followed by Tukey–Kramer post-hoc Test.

## Data Availability

The datasets used and/or analysed during the current study are accessible here: 10.25835/9cymd0ni.
